# Environmental Exposure and Mental Health in Hong Kong: Protocol for a GPS- and Biosensor-Based Observational Study

**DOI:** 10.2196/84919

**Published:** 2026-01-08

**Authors:** Yanjia Cao, Tianyu Li, Calvin P Tribby, Dorita H F Chang

**Affiliations:** 1 Department of Geography, The University of Hong Kong Hong Kong China (Hong Kong); 2 Department of Population Health, City of Hope National Comprehensive Cancer Center Duarte, CA United States; 3 Department of Psychology, The University of Hong Kong Hong Kong China (Hong Kong)

**Keywords:** digital biomarker, dynamic environmental exposure, electrodermal activity, mental health outcome, physical activity, stress response, wrist-worn biosensor

## Abstract

**Background:**

Environmental exposure, including green-blue space and walkability, may influence mental health through various physiological pathways. Traditional methods have limitations in capturing dynamic environmental exposure effects on mental health.

**Objective:**

This study aims to investigate the associations between minute-level environmental exposure and stress-related biomarkers using GPS-based measurements and wrist-worn biosensors in an urban population.

**Methods:**

In this prospective observational study, we will recruit 750 participants (aged 18-80 years) in Hong Kong from September 2024 to December 2026. This includes a pilot study supported by internal funds starting in September 2024 and external funding from the Health and Medical Research Fund by the Hong Kong Health Bureau in July 2025. Participants will undergo a 1-week experiment wearing biosensors and carrying GPS devices while completing daily surveys and entry and exit questionnaires. Primary outcomes include digital biomarkers (electrodermal activity, blood volume pulse, and skin temperature) from wrist-worn sensors and associations with the environmental exposures of air pollution, urban density, and green-blue space access. Secondary outcomes comprise questionnaire responses, physical activity metrics from accelerometer data, and environmental exposure assessments, including mobility barriers, land use mix, road accessibility, and thermal conditions. Focus groups will be conducted to refine study protocol and assess device-wearing experience.

**Results:**

The study received ethical approval from the Human Ethic Research Committee (award numbers: EA240125 and EA240514). As of June 30, 2025, a total of 150 participants completed the pilot study (September 2024 onwards), achieving a 94% (141/150) completion rate with strong device compliance (141/150, 94% valid biosensor days; 137/150, 91% daily questionnaires). Protocol refinements based on participant feedback were implemented without altering core instruments. The main study supported by the Health and Medical Research Fund, commenced in July 2025, with 30 participants enrolled as of September 2025. Data collection for 750 participants will continue until December 2026. We are at an early stage of data cleaning and preprocessing for the pilot study. Data analysis is expected to be completed by June 2027, with results anticipated to be published in 2027 and 2028.

**Conclusions:**

This study’s innovative integration of continuous environmental monitoring and stress-related biomarker may provide new insights into environment–health relationships. Findings will inform public health initiatives for mental well-being promotion and urban planning interventions.

**International Registered Report Identifier (IRRID):**

DERR1-10.2196/84919

## Introduction

The World Health Organization reported that more than one billion people worldwide experience mental health issues affecting daily lives [1]. High-rise residential buildings, particularly clustered in Hong Kong, were reported to increase the burdens of an already stressful life and threaten the well-being of residents living on the upper floors [2]. Population health surveillance data from the Hong Kong Department of Health for the years 2020 to 2022 indicated that approximately 1.2% of the surveyed population had been diagnosed with depression, while 0.9% of the respondents reported anxiety [3]. These rates were double the numbers reported from 2014 to 2015. Based on this, depression and anxiety are recognized as the most common mental health issues in Hong Kong. Furthermore, the prevalence of severe depression among Hong Kong residents increased by more than 30% between 2020 and 2023 [3]. Mental health problems present a younger trend, as a territory-wide study in 2015 showed 11.3% of children and adolescents in Hong Kong experienced common mental disorders [4]. A separate study reported an increased prevalence of 16.6% between 2019 and 2022, during social unrest and the COVID-19 pandemic [5]. However, very few people are likely to seek professional help. It has been estimated that 74% of mental health sufferers do not seek any form of professional assistance in Hong Kong [4].

Prior research has reported that the status of mental health is associated with genetic factors and wider social determinants encompassing the life span [6,7]. These determinants include socioeconomic conditions, intergenerational relations, living community social and built environment, and the affordability and accessibility of medical care [8,9]. The environment shapes many aspects of daily life. Evidence shows embracing the natural environment, especially exposure to green and blue space, contributes to improving human health [10,11]. However, existing research about environmental exposure related to the status of mental health usually focuses on the residential neighborhoods, leading to a “stationary bias” [12], while dynamic exposure assessments that concentrate on people’s mobility patterns (ie, changes in locations over time) [13] are essential to estimating total environmental exposures [14].

Traditional measurement using questionnaire surveys has been found time- and labor-consuming [15,16], and self-reported instruments are subject to individual characteristics and preferences [17,18]. Recent studies have increasingly adopted wearable devices, allowing for continuous monitoring of physiological parameters and real-time assessment of physical and mental health conditions [19-22].

**Figure 1:**
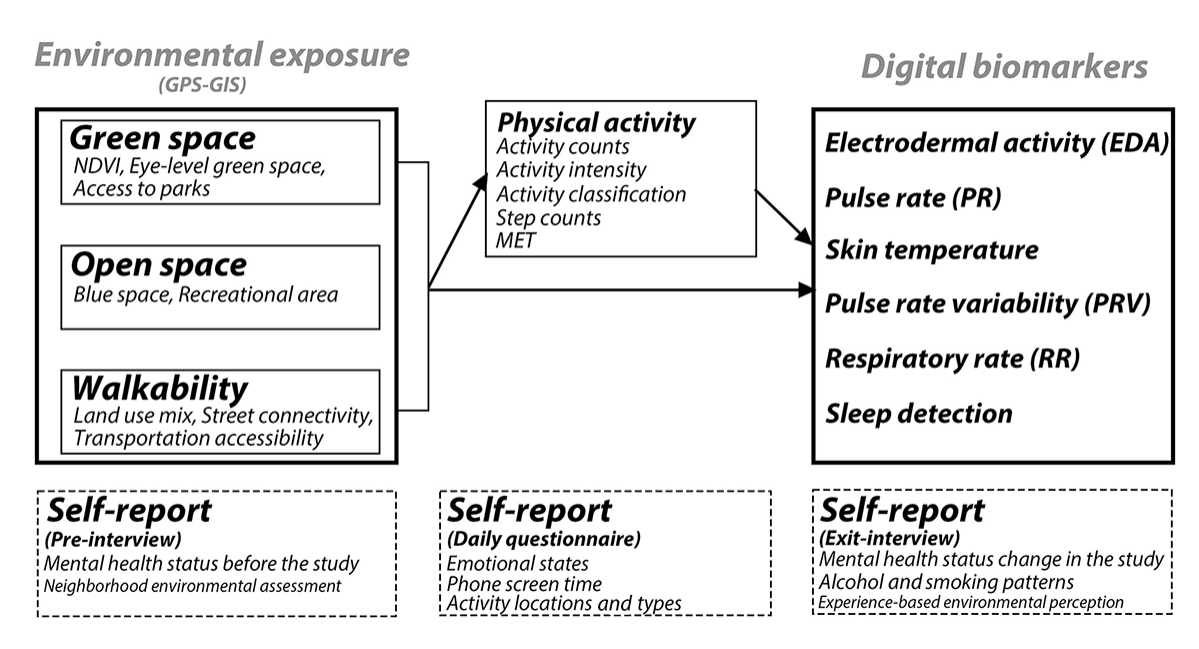
Conceptual framework of the study. GIS: geographic information system; NDVI: normalized difference vegetation index; MET: metabolic equivalent of task.

In this study, we aim to investigate the association between changes in environmental exposure, including green and blue space and walkability, and changes in biomarkers associated with mental health by using minute-level GPS-based environmental measurements and wrist-worn biosensors (Figure 1). Primary analyses will examine associations between 3 environmental exposures (air pollution, urban density, and green-blue space access) and 3 digital biomarkers (electrodermal activity, pulse rate, and skin temperature). Secondary analyses will investigate physical activity metrics and explore questionnaire responses and additional environmental assessments, including walkability, mobility barriers, land use mix, road accessibility, and thermal conditions.

We hypothesize that environmental exposure–biomarker relationships exhibit spatial and temporal heterogeneity across activity space, characterized by both acute physiological responses and cumulative effects. Physical activities and daily behavior are expected to mediate these relationships, through activity types and intensities. Moreover, we propose that the stress responses to environmental features will demonstrate individual-specific patterns, reflecting the complex interaction between personal characteristics and environmental factors. This protocol addresses current methodological gaps by implementing dynamic geospatial exposure assessment combined with continuous monitoring of digital biomarkers.

## Methods

### Study Design and Objectives

This study adopts a mixed methods longitudinal design to investigate the pathways through which environmental exposures influence mental health by physical activity mechanisms in Hong Kong adults.

Primary outcomes include digital biomarkers (electrodermal activity, pulse rate, and skin temperature) from wrist-worn sensors and their associations with the 3 environmental exposures of air pollution, urban density, and green-blue space access. Secondary outcomes comprise questionnaire responses, physical activity metrics from accelerometer data, and environmental exposure assessments, including mobility barriers, land use mix, road accessibility, and thermal conditions.

To address concerns regarding multiple testing, we will report the primary associations between environmental exposures and biomarkers defined above with α=.01 to minimize false positives (3 primary outcomes × 3 primary exposure measures = 9 comparisons). For secondary comparisons, we set α=.05, recognizing that this may include some false positives. However, these are exploratory analyses, and significant associations would be interpreted as suggestive to be examined with further study.

### Participants’ Recruitment

This study adopts a mixed methods approach, combining quantitative and qualitative methods with a cross-disciplinary approach. For sampling strategy, we apply a predefined approach by integrating stratified random sampling with multistage sampling methodologies. Initially, we categorize small territorial planning units (STPUs) in Hong Kong based on two indicators: (1) percentage of older adults (aged ≥65 years) using Hong Kong Census data: this serves as a proxy for age-specific demographic composition in each STPU, indicating areas with aging population densities and their corresponding service demands (eg, health care facilities and community support infrastructure) [23]; and (2) proximity to the nearest green space using OpenStreetMap: this indicator calculates the walking time from an STPU centroid to the nearest urban or country park, representing a crucial environmental factor for physical activities and recreation across all age groups. We further categorize STPUs into low, median, and high groups using terciles for each indicator: (1) Percentage of older adults: low (3.58%-15.78%), medium (15.78%-20.79%), and high (20.79%-65.76%); and (2) proximity to nearest park: low (8.72-56.00 minutes), medium (3.86-8.71 minutes), and high (0.10-3.85 minutes). Finally, we select STPUs based on indicator combination (percentage of older adults–proximity to green space in minutes): high–high (n=32), high–low (n=14), low–high (n=11), and low–low (n=40).

The target sample size of 750 is based on the current Hong Kong population size of 7.4 million. The sample size represents approximately 1 out of 10,000 of the population, which is a common patient representation in a mid-sized region [24,25].

The recruitment strategy includes community engagement through promotional materials in community centers and snowball recruitment through initial participants. Promotional materials include a QR code for participants to provide basic information (ie, contact, age, gender, and residential STPU). During enrollment, we perform prescreening and monitor recruitment rates across STPU strata weekly to ensure balanced distribution across demographics and geographic areas. To mitigate sampling bias toward healthier or smartphone-familiar adults, we have (1) increased targeted outreach in underrepresented areas, (2) provided device-wearing tutorials in multiple languages and formats to accommodate varying digital literacy levels, and (3) provided backup smartphones for participants without compatible devices.

### Participant Eligibility

Study participants include adults aged 18-80 years, of any ethnicity or race, who possess a valid Hong Kong identity card and have been living locally for at least 6 months. This age range is selected to provide increased exposure variability while ensuring participants maintain sufficient mobility. Adults aged >80 years are not included because of potential mobility limitations.

To be eligible, participants must be able to walk without human assistance, travel to a study visit, have a mobile phone, read and write fluently in Mandarin, Cantonese, or English, provide informed consent and comply with the protocol, and be willing and able to complete all assessments. Ineligibility criteria include (1) pregnant or nursing, (2) neuropsychological disorders or neurological illnesses that affect understanding or compliance with study protocols, (3) medical conditions that significantly limit outdoor activities or daily mobility, (4) skin conditions that may be irritated by wearing sensors, (5) inability to maintain regular device charging routines, and (6) plans to travel outside Hong Kong during the 7-day study period, or (6) conditions that prevent smartphone use for daily questionnaires.

During the enrollment process, we perform prescreening on the residential location, gender, and age groups. The number of participants from each district and the proportions of different demographic characteristics (gender and age groups) will be regularly reviewed to ensure balanced distribution and avoid oversampling from specific groups or areas.

### Pilot Data Collection Protocol Adherence

We defined feasibility thresholds to assess protocol adherence. Study completion is defined as attending both study visits (preinterview and exit interview) and providing data during the 1-week experiment period without withdrawing. We set a threshold of ≥92% completion rate. Among participants who complete the study, we set secondary compliance thresholds: (1) ≥90% of participants provide ≥5 valid biosensor days (defined as ≥18 hours/day wear time); and (2) ≥90% of participants complete ≥5 of 7 daily questionnaires. Participants who complete study visits but fail to meet these minimum data requirements will be provided 1 additional week to collect data.

### Study Procedure

**Figure 2:**
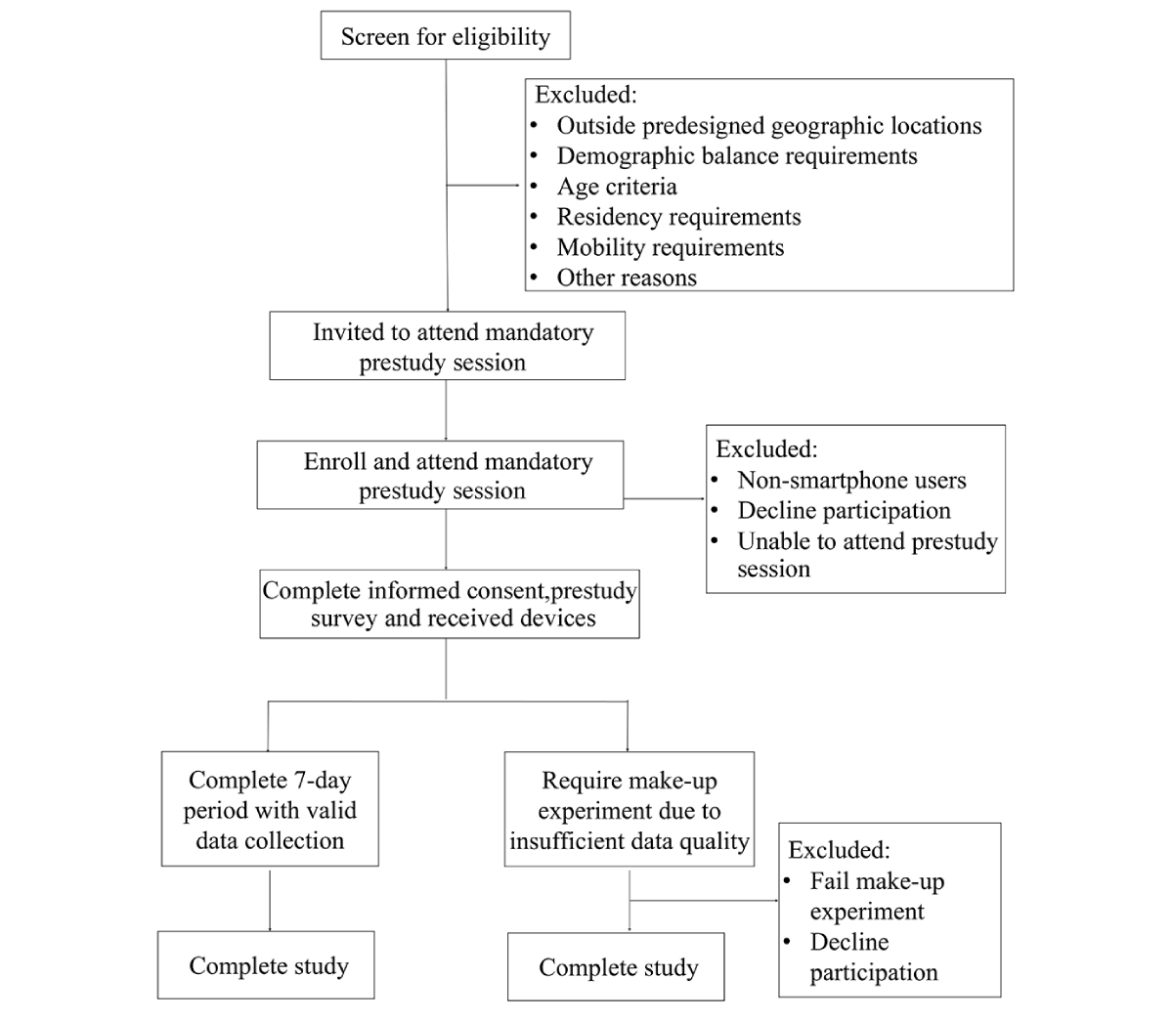
Overview of data collection process.

The overview of the data collection process is shown in Figure 2. Each participant is first invited to attend a mandatory prestudy session. During this session, participants are required to sign an informed consent form, which articulates the purpose of the study, the data collection procedure, and data confidentiality protocols. Subsequently, members of the research team provide device training for the wrist-worn biosensor (Empatica Embrace Plus) and GPS tracker (Qstarz BL 1000ST) carried in a bag or pocket. Training includes hands-on practice with proper device use, charging procedures, and a review of common issues. Participants should demonstrate competency in device operation before leaving the session. Following device training, participants are asked to complete a Short Form-12 (SF-12) Health Survey assessing their mental health over the past 4 weeks, along with their environmental perceptions and sociodemographic information. Upon completion, participants receive initial incentives of HKD $100 (US $12).

During the 7-day experimental period, participants are required to wear the biosensor for a minimum of 18 hours daily and carry the GPS tracker during all outdoor activities for at least 1 hour per day. This duration is based on prior theory indicating that valid accelerometer data requires a minimum of 3 days with at least 10 hours of daily wear time [26]. Wear time is defined as the period when the device is properly worn and collecting valid data. The biosensor uses on-skin detection algorithms to automatically identify and filter the data recorded while not being used as intended, reducing the likelihood of including unreliable observations [27]. After each day of activities, participants are asked to complete a questionnaire. Daily reminder text messages are sent to ensure questionnaire completion. In case of technical issues or device-related problems, participants are advised to contact the research team by phone for immediate assistance, including device charging support.

Upon completion of the 7-day experiment, participants are asked to complete an exit interview including: (1) the SF-12 questionnaire to assess their mental health during the study week, (2) an exit survey covering study feedback and retrospective behavior, and (3) a questionnaire assessing their perceived built environment based on their daily activity spaces. Participants who can successfully provide at least 5 days of data from both biosensor and GPS devices could receive full incentives of HKD $400 (US $50). If data quality does not meet the criteria, a one-time make-up experiment is offered.

### Digital Biomarkers Associated With Mental Health

Participants’ mental health is measured by a wrist-worn biosensor, a physiological monitoring device that captures an individual’s motion-based activity, blood volume pulse (BVP), and skin temperature and links them to physiological signals (Table 1). Skin conductance is measured in microsiemens (μS) and ranges from 2-100 μS. The sampling rate is 4 Hz. Electrodermal activity (EDA) explorer will be used to detect peaks in 5-second periods of the clean electrodermal 5-minute signal. The biosensor also assesses acceleration and rotation (actigraphy) and BVP by photoplethysmography (PPG) at sampling rates of 64 Hz, as well as peripheral (skin) temperature at sampling rates of 1 Hz.

**Table 1:** Measurements for raw sensor data used in this study and their relationships with mental health.

High-frequency sensor data	Measurement method	Sampling rate	Pathway to mental health outcomes	Connection to mental health outcomes
EDA^a^	Skin conductance	4 Hz	Sympathetic nervous system activation	Higher EDA indicates increased stress and emotional arousal
BVP^b^	PPG^c^	64 Hz	Cardiovascular regulation	Irregular patterns reflect anxiety and stress states
Skin Temperature	Infrared sensor	1 Hz	Peripheral vasoconstriction	Decreased peripheral temperature indicates sympathetic nervous system activation during stress
Activity (actigraphy)	3-axis accelerometer	64 Hz	Physical activity patterns	Regular physical activity promotes positive mental health through various physiological and psychological pathways

^a^EDA: electrodermal activity.

^b^BVP: blood volume pulse.

^c^PPG: photoplethysmography.

In subsequent analysis, these raw sensor data will be processed into minute-level digital biomarkers through Empatica’s proprietary algorithms, which have been validated through prospective clinical studies sponsored by Empatica [28,29]. The digital biomarkers are aggregated into 1-minute windows, including EDA (0.01-100 μS), pulse rate (24-240 beats per minute [bpm]), skin temperature (28 °C-35 °C) as primary outcomes, as well as pulse rate variability (PRV, root-mean-square of successive difference [RMSSD] up to 300 ms), and respiratory rate (6-60 breaths per minute) as secondary outcomes. Sleep detection is also monitored, with periods categorized as awake, sleep, or interruption. All these digital biomarkers will be computed and aligned at 1-minute intervals using specific aggregation methods for each indicator.

Individual baseline values are measured in two phases. First, during the preinterview session, participants wear the biosensor device while completing preinterview survey, which typically takes more than 10 minutes in a quiet environment. These measurements during the survey completion period serve as their initial baseline. Second, during the study period, we identify the daily baseline for each participant by selecting the 15-minute continuous period when their EDA levels are at their lowest, given that EDA is a primary indicator of sympathetic nervous system activation and stress response [30]. During these periods, we also record corresponding pulse rate, respiratory rate, and skin temperature values. These daily baseline values will be averaged across multiple days to establish a personalized baseline.

### Physical Activity and Mobility Measurements

Physical activities are measured using accelerometer data from both biosensor and GPS devices. The biosensor device captures raw accelerometry data through its integrated 3-axis accelerometer at 64 Hz sampling rate. Using proprietary machine learning algorithms, the accelerometry signals are processed into minute-level digital biomarkers, including activity counts (0-200 counts), activity intensity levels (categorized as sedentary, low, moderate, or vigorous), movement classification (still, generic, walking, or running), step counts (up to 192 steps), and metabolic equivalent of task (MET, 0.95-8.53).

Raw GPS data are collected with 1-second sampling intervals. Table 2 listed each measurement, sample resolution, and aggregation process. The GPS device logs geographic coordinates, distance, speed, elevation, and time. In subsequent analysis, GPS data (1-second intervals) will be aligned with the biosensor data at 1-minute intervals. Within each 1-minute window, all GPS location points will be retained rather than aggregating to a single point. This approach maintains detailed movement patterns and allows precise assessment of environmental exposures.

**Table 2:** Measurement of digital biomarkers and location indicators at 1-minute resolution.

Indicator	Raw sampling	Aggregation method	Output interval	Range	Resolution
Activity counts	64 Hz (accelerometer)	Zeros-crossing count in filtered data	1 minute	0-200 counts	1 count
Activity intensity	Derived from MET^a^	MET threshold categorization	1 minute	4 categories (sedentary, low physical activity, moderate physical activity, or vigorous physical activity)	Categorical
Activity classification	64 Hz (accelerometer)	3-second nonoverlapping windows aggregation	1 minute	4 categories (still, generic, walking, or running)	Categorical
Step counts	64 Hz (accelerometer)	Step detection from accelerometer data	1 minute	0-192 steps	1 step
EDA^b^	4 Hz	Average over window	1 minute	0.01-100 μS	~55 pS
MET	64 Hz (accelerometer)	Energy expenditure calculation	1 minute	0.95-8.53 MET	—^c^
Pulse rate	64 Hz (PPG^d^)	Aggregation of 10-second windows	1 minute	24-240 bpm^e^	1 bpm
PRV^f^ (RMSSD^g^)	64 Hz (PPG)	RMSSD calculation from interbeat intervals	1 minute	0-300+ ms	0.000001 ms
Respiratory rate	64 Hz (PPG)	Combining 30-second windows	1 minute	6-60 brpm^h^	1 brpm
Skin temperature	1 Hz	Average over window	1 minute	0 °C-85 °C	0.01 °C
Sleep detection	Derived from activity counts	Activity analysis for sleep/wake periods	1 minute	0 (awake), 101 (sleep), 102 (wake), 300 (interruption)	Categorical
GPS location data	1 second	Total area covered by multiradius buffers around sequential points within 1-minute window	1 minute	0-1 km²	0.0001 km²

^a^MET: metabolic equivalent of task.

^b^EDA: electrodermal activity.

^c^Not applicable/Not available.

^d^PPG: photoplethysmography.

^e^bpm: beats per minute.

^f^PRV: pulse rate variability.

^g^RMSSD: root-mean-square of successive difference.

^h^brpm: breaths per minute.

### Environmental Exposure Measurements

We will measure the built environment from multiple perspectives. Primary environmental exposures include air pollution (particulate matter ≤2.5 µm [PM2.5], nitrogen dioxide [NO_2_], and air quality index [AQI]), urban density (ground coverage ratio [GCR] and street coverage ratio [SCR]), and green-blue space access (normalized difference vegetation index [NDVI] and eye-level exposure metrics). Secondary environmental assessments comprise mobility barriers, land use mix, road accessibility, and thermal environment.

Air pollution exposure will be evaluated by PM2.5, NO_2_, and AQI from Hong Kong’s 18 air quality monitoring stations operated by the Environmental Protection Department. Kriging or inverse distance weighting methods will be applied to estimate air pollution levels across the study area. Urban density will be quantified using GCR and SCR for each trajectory buffer area. GCR represents the ratio of total building footprint to buffer area, while SCR indicates the percentage of vehicular street coverage. Green-blue space exposure will be quantified using multiple methods. Greenspace availability will be measured by NDVI from Sentinel-2 satellite imagery (10-meter resolution). Eye-level exposure to green space, recreational areas, blue spaces, and public spaces will be evaluated using Google Maps Street view images [31,32], which will be newly acquired during the analytical process in 2026.

For secondary environmental assessments, mobility barriers will be assessed by analyzing street intersection density, obstacles, slopes, surface conditions, traffic black spots (ie, high-risk areas for accidents), and barrier-free facilities (eg, ramps, staircases, footways, lifts). These factors will be derived from government infrastructure data and 3D pedestrian network datasets within a defined radius of each GPS position. Land use mix will be quantified by the Shannon entropy index [33] from government land use raster files. Point of interest (POI) density will be computed through kernel density estimation of geocoded points from government POI databases. Road accessibility will be calculated using degree, closeness, and betweenness centrality measures will be derived from graph theory algorithms [34]. Furthermore, the thermal environment will be assessed through land surface temperature, which will be retrieved from Landsat 8 thermal infrared sensor bands using the split-window algorithm and atmospheric correction techniques.

Dynamic environmental exposure will be calculated using kernel density estimation (KDE) at multiple temporal scales based on GPS trajectories [35,36]. Specifically, we will analyze exposure at the minute level, daily level, and weekly level. This multiscale approach allows us to examine both acute and cumulative environmental exposures in relation to physical activity patterns and stress responses. It distinguishes between acute high-intensity exposures at the minute level and sustained low-intensity exposures over longer periods, which may produce equivalent cumulative values but represent distinct exposure patterns across the study period. The KDE-derived exposure surface will be integrated with environmental variables to create composite exposure metrics, which will then be temporally aligned with accelerometer-derived activity data and physiological measurements. For example, minute-level environmental exposure can be directly linked to concurrent physical activity intensity and MET values, while daily and weekly exposure metrics can be associated with aggregate activity patterns and cumulative physiological responses.

For the overall measurement of built environment characteristics and exposure metrics, we will perform index normalization across all environmental indicators, as these measures are originally on different scales (eg, NDVI ranges from –1 to 1, while AQI ranges from 0 to 500). Each environmental indicator will be transformed to a standardized scale (0-1) while maintaining their relative distributions, supporting both within-subject comparisons of different environmental exposures and between-subject comparisons of exposure patterns.

### Self-Report Measures and Covariates

In addition to objective measurements (eg, GPS-recorded location and biosensor-derived biomarkers), subjective measurements were collected through structured questionnaires at 3 time points: pre-experiment, during-experiment, and postexperiment. The questionnaires are provided in both English and Cantonese, allowing participants to choose their preferred language.

The preinterview questionnaire contained health-related quality of life assessment using SF-12, which evaluates both physical and mental health components, health behaviors including smoking and alcohol consumption patterns, and perceived built environment quality. The built environment perception was assessed through 3 domains regarding public service accessibility, public open space and green space quality, and transportation convenience. These items were measured on a 5-point Likert scale from 1 (strongly disagree) to 5 (strongly agree). Weather perception and comfort assessment were also included.

During the experiment, participants completed daily surveys recording their travel modes, activity locations and types, estimated phone screen time, and clothing combinations. Emotional states were assessed using a modified version of the Positive and Negative Affect Schedule, comprising 10 mood items rated on Likert scales [37].

At the end of the experiment, participants completed an exit interview reflecting on their experiences over the 7-day experimental period. The interview assessed mental health using the Patient Health Questionnaire for depression symptoms, and health behaviors through detailed alcohol consumption and smoking patterns (frequency, quantity, and motivations). Environmental perceptions and mood responses to different environments were evaluated, including emotional responses to various urban settings such as public gardens, waterfront promenades, urban streets, and mountain trails. Overall device experience and weather comfort preferences were also assessed. These subjective measurements complement our objective data by capturing perceptions and experiences that cannot be recorded by GPS devices or biosensors.

Demographic covariates include age, gender, height (cm), weight (kg), education level, marital status, occupation status, housing type, length of residence in Hong Kong (years), living conditions, monthly household income (excluding welfare or disability allowances), household size (including the participant but excluding foreign domestic workers), residential area in Hong Kong, and building of residence.

### Data Processing and Analysis

GPS data (recorded at 1-second intervals) and physiological data from the biosensor will be temporally aligned at 1-minute intervals. To explore the relationship between environmental exposure and mental health, descriptive statistics will be computed for all variables, including biomarkers and demographic characteristics collected from biosensors and survey data. Diagnostic statistics will be applied to assess model adequacy. Outcome variables (eg, biomarkers) will be log-transformed when necessary to better approximate Gaussian distribution for residuals.

Environmental exposure will be quantified by applying kernel weights to the GPS data. This method accurately measures the precise exposure at the GPS point level, with a proximity-weighted raster surface at fine granularity (50 m × 50 m) will be constructed for the activity space of each individual [38]. For each grid cell, exposure values will be calculated based on kernel density estimation, with weights proportional to the time spent in each location.

To analyze the relationship between these environmental exposures and physiological responses, we will use multiple analytical approaches. Statistical testing will follow a hierarchical approach with α=.01 for primary analyses examining the 9 prespecified associations (3 environmental exposures × 3 digital biomarkers) and α=.05 for secondary analyses. Generalized linear mixed models will serve as our primary method to examine the direct relationships between GPS-derived exposure measures and biomarker variations, accounting for individual differences through random effects. To capture potential nonlinear relationships, we will use generalized additive mixed models. These models will be enhanced with time-series analysis techniques, including autoregressive integrated moving average models to examine temporal patterns and cross-correlation functions to identify potential lag effects between environmental exposure and physiological responses. This analytical framework allows us to understand both the immediate and delayed effects of environmental exposure on physiological responses, while accounting for individual variations and temporal dynamics.

Structural equation modeling will be used to investigate the mediating role of physical activities between environmental exposure and mental health. It accounts for both observable and latent variables, particularly mental health as measured by the SF-12 questionnaire. To further explore the spatiotemporal variation in the relationship between environmental exposures and mental health changes, geographically and temporally weighted regression will be used, which extends traditional geographical weighted regression by incorporating both spatial and temporal dimensions, revealing the varying relationships across space and time. This approach captures dynamic patterns of environmental exposure influences on mental health status across different locations and temporal periods.

### Ethical Considerations

The Human Ethics Research Committee at The University of Hong Kong approved the study procedures (award numbers: EA240125 and EA240514). Each participant provided written informed consent before the start of the experiment. Participants received an initial incentive of HKD $100 (US $12) and, upon completing the experiment, the remaining incentive of HKD $400 (US $50).

As we are collecting data with GPS locations, biomarkers, and questionnaires with personal demographic information, the sensitivity of this dataset requires high protection. The study was approved by the Human Research Ethic Committee at the University of Hong Kong. Only staff and student members listed on this ethics approval will have temporary access to study participants’ names. This information will be pseudo-anonymized for subsequent data analysis. Student helpers for data collection were all required to sign a data protection and confidentiality agreement. The data will be stored in a password-protected computer and folder managed by the corresponding author throughout the project period. Due to the large quantity of data, the corresponding author has secured access to a high-performance computing account hosted by the University of Hong Kong. All raw data will be backed up daily. All other data and codes for analytical methods can be obtained by contacting the corresponding author. The primary disseminations of our research results will comprise journal publications and presentations at academic conferences. We will also generate reports and provide evidence-based materials for policymakers to facilitate the development of more effective mental disorder intervention strategies.

## Results

This study received internal funding in May 2024 and external funding from the Health and Medical Research Fund of Hong Kong Health Bureau in July 2025. The Human Ethics Research Committee at The University of Hong Kong approved the study procedures (award numbers: EA240125 and EA240514). Each participant provides written informed consent before participating in the study.

This pilot study supported by internal funds began in September 2024. The initial setup and testing of GPS devices and biosensors were conducted from October to December 2024. As of June 30, 2025, a total of 150 participants from this pilot study had completed the 1-week experiment period, including participants from diverse population groups such as older adults and foreign domestic workers. This exploratory study aimed to validate the data collection protocol and refine study procedures, particularly focusing on device-wearing compliance and data quality assurance. As a pilot feasibility study, we monitored these feasibility metrics during data collection. We achieved a 94% (141/150) study completion rate, with participants providing ≥5 valid biosensor days and 91% (137/150) completing ≥5 of 7 daily questionnaires. These observations demonstrate the feasibility of our protocols for the larger-scale investigation.

Participant feedback sessions for protocol refinement were conducted during exit interviews throughout the pilot study period, helping identify key challenges in data collection procedures and device usage. Some participants reported difficulty with data collection during the summer months due to high temperatures and extreme weather conditions, which resulted in limited outdoor activity time and consequently reduced GPS recording coverage. A portion of participants found it challenging to maintain consistent device-wearing time, particularly during sleep or water-related activities such as swimming or showering. Several participants also noted that the limited battery life of the biosensors required frequent charging, occasionally leading to data gaps. Based on these findings, the research team has implemented enhanced support protocols with regular check-ins and reminders, detailed device management guidelines, and flexible data collection scheduling. These refinements did not change the core data collection instruments or measures, ensuring comparability between pilot and main study data.

Following the successful pilot phase, the main study, supported by the Health and Medical Research Fund, commenced in July 2025, with 30 participants enrolled and the protocol implemented as of September 2025. Full data collection for 750 participants will continue until December 2026, followed by comprehensive data analysis focusing on the associations between environmental exposure, physical activity, and mental health biomarkers. Environmental exposure data and physiological measurements are being continuously collected and processed, with specific attention to seasonal variations and weather conditions that may affect data quality. We are at an early stage of data cleaning and pre-processing for the pilot study. Data analysis is expected to be completed by June 2027, with results anticipated to be published in 2027 and 2028.

## Discussion

### Anticipated Findings

We expect to observe improved PRV and reduced EDA when exposed to green and blue spaces, with greater exposure duration and intensity associated with greater effects on these biomarkers. Conversely, we anticipate that higher air pollution levels (PM2.5, NO_2_, and AQI) and greater urban density (higher GCR and SCR) will be associated with elevated EDA and reduced PRV. This study will examine the temporal dynamics of these effects through continuous monitoring and identify the optimal duration and intensity of environmental exposures for stress reduction. We predict cumulative effects of environmental exposure on digital biomarkers associated with mental health over the study period. Participants with higher cumulative exposure to green spaces may exhibit lower baseline stress levels and better stress recovery patterns, while those with long-term exposure to air pollution and high urban density may show elevated baseline stress and impaired recovery.

Additionally, our minute-level assessment examines whether physical activity mediates the relationship between environmental exposure and stress reduction. We anticipate that physical activity, operationalized through MET, will partially mediate this relationship. Specifically, we expect moderate-to-vigorous physical activity during green-blue space exposure to strengthen the associations with lower stress levels.

Furthermore, we expect the associations between all 3 primary environmental exposure and stress biomarkers to vary across demographic subgroups and baseline mental health status. In particular, older adults and individuals with elevated baseline stress may exhibit distinct biomarker responses to environmental exposure compared to younger adults and those with lower baseline stress. As part of our secondary analyses, we will also explore associations between secondary parameters (mobility barriers, land use mix, road accessibility, and thermal environment) and additional stress-related biomarkers such as respiratory rate and sleep quality.

### Comparison to Prior Work

This study addresses critical gaps in existing literature through several methodological innovations. Existing studies on mental health have adopted the activity and travel logs to record daily activities [39,40]. However, the locational accuracy of these records is relatively low [41]. Compared to travel logs, GPS tracking, with continuous location information, captures environmental exposure at a more precise spatial and temporal granularity and has been widely used in public health [42,43]. For example, using ecological momentary assessment, researchers have examined the association between momentary depressive symptoms and mobility-based microurban environmental exposure by integrating GPS and accelerometer data to track daily routines [44-47]. While GPS has been widely used in public health research, there remains limited work that combines GPS data with objective measures of urban environments, including urban density, land use mix, and accessibility to facilities, to investigate their impacts on mental health [48,49]. Moreover, measurement on mental health is limited at low temporal resolution with self-reports [15,16]. Our minute-level assessment fills this gap by allowing for precise evaluation of associations between environment, physical activities, and biomarkers associated with mental health.

Previous literature has indicated that exposure to green space is able to mitigate depressive symptoms, alleviate anxiety [50], and stress, and improve cognitive abilities [51]. In addition to the visual characteristics of the environment, practical aspects—particularly the environment’s walkability—have been linked to improved mental health. Walkability includes different aspects of the built environment, such as land use mix, intersection density, and public transportation accessibility [52,53]. Walkability has also been linked to higher intensity of physical activity and more social interaction [54]. Therefore, our subsequent research papers will have a focus on the impact from built environment on fluctuations in mental health status.

Wearable technology has the potential to revolutionize mental health care systems by continuously monitoring vital physiological parameters, allowing real-time assessment [20,21]. Physiological parameters, including heart rate variability (HRV), EDA, and behavioral parameters captured by the wearable biosensors, have already demonstrated encouraging results in the identification of stress, anxiety, and depression [55]. For example, using such wearables, it has been shown that short-term exposure to urban green spaces can lead to lower blood pressure, improved HRV indices, and potentially reduced heart rate, indicating a decrease in stress levels [56]. In terms of stress mechanisms, EDA reflects sympathetic nervous system activity, indicating emotional arousal, while skin temperature fluctuations, mediated by vasomotor responses, reveal stress-induced autonomic changes [57]. Respiratory rate, HRV, along with heart rate variations can all reflect stress-related physiological responses.

However, existing studies using biomarkers to link environmental exposure and health outcomes have been limited by insufficient sample size, with participant numbers typically below 20 [36]. These studies often measured immediate physiological responses and acute stress markers during outdoor environmental exposure [58,59], without investigating cumulative effects. The absence of baseline measurements before environmental exposure in many studies prevents accurate assessment of stress-related changes. [60]. This study overcomes these limitations by implementing continuous monitoring over extended periods, establishing baseline measurements, and recruiting a larger and more diverse sample. We investigate both acute and cumulative environmental exposures and their contributions to mental health outcomes through routine residential exposure and daily activity patterns over time. This approach is particularly important since environmental stress in disadvantaged neighborhoods may serve as a mechanism underlying health disparities [61,62].

### Strengths and Limitations

This study has several strengths. First, the integration of minute-level GPS tracking with continuous biosensor monitoring represents a significant methodological advancement, allowing precise spatiotemporal assessment of environmental exposure and stress-related responses. Second, we are collecting data from diverse population groups, including older adults and foreign domestic workers, allowing us to compare demographic groups (eg, younger adults vs older adults) and explore differences in stress impact from physical-social-built environments. This diversity enhances the generalizability of our findings across different population segments in Hong Kong. Third, the combination of objective biomarkers, questionnaires, and GPS records provides a comprehensive assessment of the relationship between environmental exposure and mental health. Fourth, the extended monitoring period allows for examination of both acute and cumulative effects of environmental exposure, addressing a significant gap in existing literature. Lastly, this study design includes baseline measurements before environmental exposure, enabling accurate assessment of stress-related changes.

However, several limitations should be acknowledged. First, our exclusion criteria (no neuropsychological disorders, no severe mobility limitations, able to maintain device charging) may systematically exclude individuals at high mental health risk, who may be less likely to enroll in the study. This limits the generalizability of findings to the broader population with mental health concerns. Second, the study protocol cannot be applied for children and teenagers, who have become a major concern in the region regarding mental health problems. As teenagers and children require a guardian when conducting the experiment, different study protocols are suggested for future research. Third, participant reactivity to wearing monitoring devices may influence behavior and stress-related responses, although this effect typically diminishes over time. Lastly, this protocol is not designed as a randomized controlled trial, and therefore is not applicable for causal inference on environmental exposure.

### Feasibility Challenges

Several challenges were encountered during pilot data collection. First, data collection was difficult during the summer time due to the high temperatures or extreme weather conditions, resulting in limited outdoor activity time and thus limited GPS recording. Although subsequent studies could investigate seasonal difference or thermal influence on mental health status, recommendation for data collection shall be considered under normal weather conditions. Second, some participants had difficulty maintaining consistent device-wearing time, particularly during sleep or water-related activities (such as swimming or showering). Third, the battery life of biosensor required regular charging, occasionally leading to data gaps.

In addition, different participant subgroups presented distinct challenges. Older adults (aged >65 years) required longer onboarding sessions (average 45 minutes vs 30 minutes) and reported concerns about device loss when wearing the device outdoors. Foreign domestic workers had limited private time for device charging and questionnaire completion, typically available only late evening, and experienced scheduling constraints due to work commitments. Initial contact-to-enrollment conversion rate was 68%, with time commitment cited as the primary barrier. These population-specific barriers informed adjusted support strategies, including flexible appointment scheduling and charging solutions.

The research team implemented adjusted support strategies. For general operational issues, regular check-ins and detailed guidelines were provided throughout the study procedure, and potential support from backup devices was made available to resolve charging-related data gaps. For population-specific barriers, tailored solutions included flexible appointment scheduling, instructional videos for older adults, and portable charging options.

### Next Steps and Future Directions

Full data collection with our target sample of 750 participants is expected to be completed by December 2026. Our subsequent analysis will explore the impact of daily activities and environmental exposure on both sleep quality and stress recovery patterns in urban environments. We will examine the mediating role of physical activity in the relationship between environmental exposure and mental health biomarkers.

Building on this protocol, future research will leverage the Convolutional Neural Networks–Long Short-Term Memory hybrid model to develop real-time stress prediction systems that forecast biomarker responses based on individuals’ environmental exposures and physical activities. Random Forests and Gradient Boosting Machines will be used to identify critical environmental features and exposure thresholds that trigger stress reduction, allowing personalized exposure recommendations. We will develop and test targeted interventions based on identified environmental features, to transform findings into actionable mental health promotion strategies. We anticipate contributing to evidence-based urban design and public health policies that promote mental well-being in dense urban environments.

### Dissemination Plan

We will disseminate findings through multiple channels to ensure broad impact. At the academic level, we will publish in open-access and high-impact journals, present at conferences, and share findings through research seminars and a quarterly research exchange platform. For policy and community impact, we will collaborate with Hong Kong Health Bureau to incorporate evidence-based recommendations into official mental health guidelines and share findings with therapists to inform personalized intervention strategies. Analytical code will be made available in public repositories upon publication to facilitate reproducibility of similar research in the future.

## Data Availability

The datasets generated from this research are not publicly available due to the need to protect participant privacy. But they are available from the corresponding author upon request with justification.

## Abbreviations

AQI: air quality index
bpm: beats per minute
BVP: blood volume pulse
EDA: electrodermal activity
GCR: ground coverage ratio
HRV: heart rate variability
KDE: kernel density estimation
MET: metabolic equivalent of task
NDVI: normalized difference vegetation index
NO2: nitrogen dioxide
PM2.5: particulate matter ≤2.5 µm
POI: point of interest
PPG: photoplethysmography
PRV: pulse rate variability
RMSSD: root-mean-square of successive difference
SCR: street coverage ratio
SF-12: Short Form-12
STPU: small territorial planning unit
